# *Trichosomoides nasalis* (Nematoda: Trichinelloidea) in the murid host *Arvicanthis niloticus:* Migration to the epithelium of the nasal mucosa after intramuscular development

**DOI:** 10.1051/parasite/2012194359

**Published:** 2012-11-15

**Authors:** E.H. Fall, M. Diagne, C. Martin, Y. Mutafchiev, L. Granjon, K. Ba, K. Junker, O. Bain

**Affiliations:** 1 Département de Biologie Animale, Faculté des Sciences et Techniques, UER 201/ESP 2014, Université Cheikh Anta Diop de Dakar BP5005 Dakar Sénégal; 2 UMR 7245 MCAM MNHN CNRS & UMR 7205 OSEB MNHN CNRS, Muséum National d’Histoire Naturelle 61, rue Buffon CP52 75231 Paris Cedex 05 France; 3 Institute of Biodiversity and Ecosystem Research, Bulgarian Academy of Sciences 2 Gagarin Street 1113 Sofia Bulgaria; 4 IRD, UR 022 CBGP-Bel Air BP 1386 Dakar CP 18524 Sénégal; 5 ARC-Onderstepoort Veterinary Institute Private Bag X05 Onderstepoort 0110 South Africa

**Keywords:** *Trichosomoides*, *Trichinella*, rodent, migratory route, mating site, *Trichosomoides*, *Trichinella*, rongeur, voie migratoire, site d’accouplement

## Abstract

Knowledge of the biology of the trichinelloid subfamily Trichosomoidinae is poor. *Trichosomoides nasalis* is a common parasite of *Arvicanthis niloticus* (Muridae) in Senegal, and a procedure for experimental infections has been established. It has been demonstrated that larvae develop in striated muscle fibres, similar to *Trichinella* spp., but they are not arrested in the first stage, and they reach the adult stage within three weeks. In the present histological study it is shown that *T. nasalis* females and dwarf males migrate from the abdomen and thorax to the host’s muzzle, moving through connective tissues and between muscles. A few migrating specimens were also found in the blood vessels of the nasal mucosa. While sexes were still separated in the lamina propria of the mucosa, females recovered from the epithelium contained intra-uterine males. Worms were found between the incisors in the mucosa of the anterior and median conchae which are rich in mucous cells. Only the pseudostratified epithelium was parasitized. Under natural conditions, the inflammation of the nasal mucosa that is induced by the parasites might reduce the competitiveness of infected rodents when foraging or looking for potential mates.

## Abstract (fr)

La biologie de la sous-famille des Trichosomoidinae est peu connue. *Trichosomoides nasalis* est un parasite commun d’*Arvicanthis niloticus* (Muridae) au Sénégal, et une procédure d’infestation expérimentale a été mise au point. Il a été démontré que les larves se développent dans les muscles striés, comme pour *Trichinella* spp, mais elles ne s’arrêtent pas au premier stade et atteignent le stade adulte en trois semaines. Cette étude histologique montre que les femelles et des mâles nains de *T. nasalis* migrent depuis l’abdomen et le thorax jusqu’au museau de l’hôte, et se déplacent à travers le tissus conjonctif entre les muscles. Quelques specimens en migration ont également été observés dans les vaisseaux sanguins de la muqueuse nasale. Si les sexes sont toujours séparés dans le lamina propria de la muqueuse, des mâles intra-utérins sont observés chez des femelles au niveau l’épithélium. Au niveau nasal, des vers ont été trouvés entre les cornets antérieurs et médians riches en cellules muqueuses. Seul l’épithélium pseudostratifié est parasité. En condition naturelle, l’inflammation de la muqueuse nasale provoquée par les parasites pourrait réduire la capacité des rongeurs infectés dans leur recherche de nourriture ou de partenaires.

## Introduction

*Trichosomoides nasalis* Biocca & Aurizi, 1961 is a trichinelloid nematode of which the females that contain dwarf males in their uteri, live in the epithelium of the nasal mucosa of their rodent host (Diagne *et al.*, 2004; see erratum at the end of this paper). It is a common parasite of the murid *Arvicanthis niloticus* (Geoffroy) in Senegal (Diagne *et al.*, 2000). A laboratory-based breeding programme for this host has been established, which made it possible to study the biology and transmission of *T. nasalis*, a species in the rarely studied group, the Trichosomoidinae (Anderson, 2000). It was recently discovered that larval development of *T. nasalis* occurs in the striated muscle fibres of the rodent. This highlighted similarities with the *Trichinella* spp., suggesting that the muscular larval phase might be a primary feature in the Trichosomoidinae, although this was not previously suspected (Fall *et al.*, 2012). However, the development of the *Trichinella* spp. is arrested at the end of the first stage (Kozek, 1971), whereas in *T. nasalis* all four larval stages occur in the muscle fibres of the abdominal and thoracic walls whereafter migration to the nasal mucosa takes place (Fall *et al.*, 2012). The aim of this study was to elucidate the late migratory route from the muscles to the nasal mucosa and to determine the mating site of *T. nasalis*.

## Materials and Methods

**Fig. 1.**
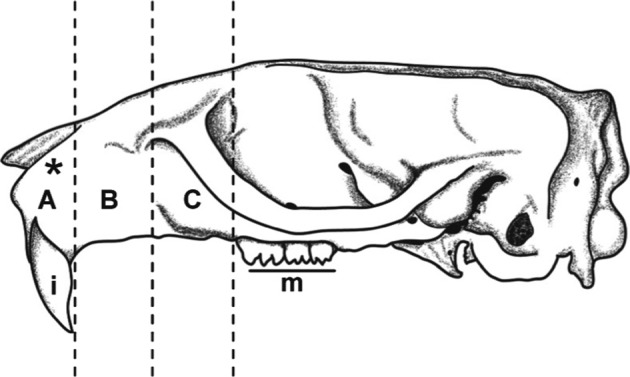
The three maxillar regions delineated for histology, shown on a rodent skull in lateral view. A, from the vestibulum to the beginning of incisor (i); B, incisor tooth region; C, from the posterior part of the incisor to the first molar (m). * indicates the position of adult *Trichosomoides nasalis*.

Thirty *A. niloticus* were experimentally infected by one or two intraperitoneal injections, as described by Fall *et al.* (2012). Infected rodents were kept isolated in order to avoid any uncontrolled contamination with the parasite. Rodents were euthanized from 19-21 days post-infection (dpi) which corresponds to the period of migration of worms to the nasal mucosa, as established by Fall *et al.* (2012). The thoracic wall and the maxilla were fixed in 10 % formalin as described by Diagne *et al.* (2004), and subsequently decalcified for three hours (rapid decalcification with DC-LMR®). The thorax was divided into four parts, the maxilla into three parts, as determined on the basis of yet unpublished observations, that adult *T. nasalis* were usually recovered from a specific site in the maxilla, namely between the roots of the incisors. Thus, three frontal pieces, A, B and C, each approximately 0.5 cm thick, were cut from anterior to posterior (Fig. 1). These tissue samples were embedded in paraffin wax, sectioned at 5 μm and stained with Mayer’s haemalum and eosin.

## Results

**Fig. 2.**
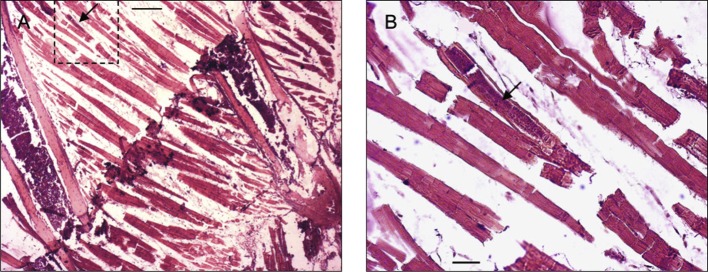
*Trichosomoides nasalis* in a thoracic muscle fibre. A, overview of ribs and musculature, parasite (arrow) within marked area. B, close-up of infected fibre. Arrow indicating parasite. Scale bars in μm: A, 400; B, 50.

**Fig. 3.**
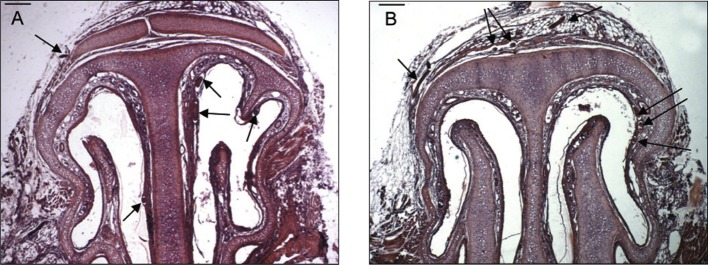
Two frontal sections of the nasal cavities at the level of the muzzle, showing the conchae projecting into the nasal cavities. A, on the left the median concha, and on the right the anterior and median conchae are visible. Arrows indicate the localization of five *Trichosomoides nasalis*. B, slightly posteriorly, only the median conchae are visible in the two cavities. Arrows indicate the localization of seven *T. nasalis*. Scale bars in μm: A, B, 200.

**Fig. 4.**
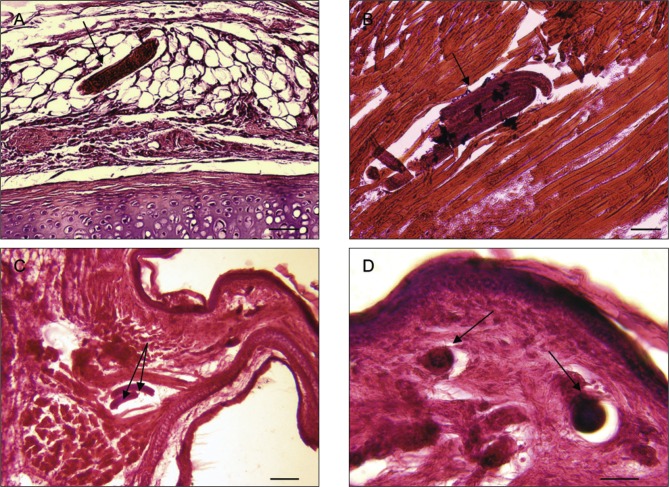
*Trichosomoides nasalis* (arrows) in tissues external to the nasal cavities. A, in the adipose tissue of the dorsal region of the muzzle. B & C, between muscle fibres of the nasal muzzle and vestibulum, respectively. D, in dermal connective tissue of the nasal vestibulum, two worms in transverse section. Scale bars in μm: A, B, D, 50; C, 100.

**Fig. 5.**
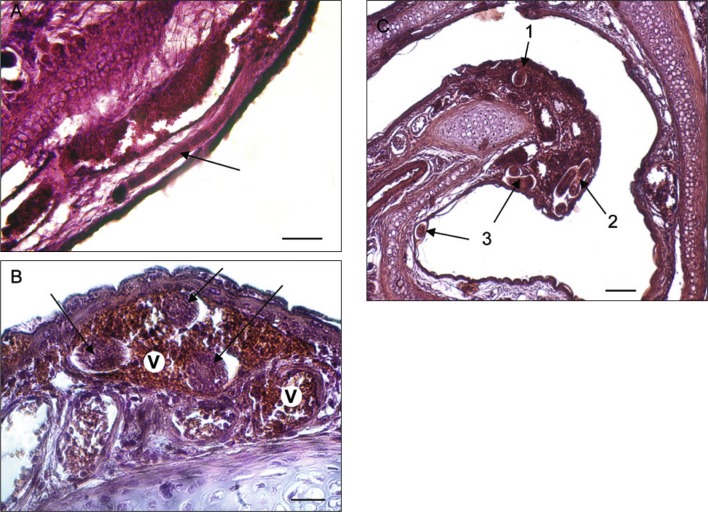
*Trichosomoides nasalis* (arrows) in tissues of the nasal cavities. A, in the lamina propria, a worm in longitudinal section, at level of stichocytes. Note the large longitudinal vessel below, and another worm on left in the connective tissue. B, worm in a blood vessel of the lamina propria (V = blood vessel). C, four worms situated around the median concha, in the lamina propria (1), a blood vessel (2) and the pseudostratified epithelium (3), respectively. Scale bars in μm: A, 75; B, 30; C, 100.

**Fig. 6.**
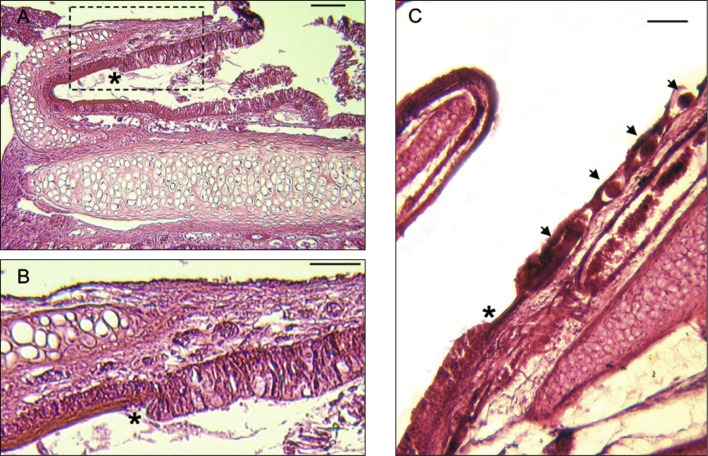
*Trichosomoides nasalis* in the pseudostratified epithelium. A, the two types of epithelia of the anterior concha: epithelium with mucous cells and pseudostratified epithelium without mucous cells but with keratinized upper lining. B, marked area in A enlarged to show detail of contact between the two epithelia. C, *Trichosomoides nasalis* female in the pseudostratified epithelium. Note the large longitudinal blood vessel below. * Marks contact between pseudostratified epithelium (on the right, with a parasite indicated by arrows) and epithelium with mucous cells. Scale bars in μm: A, C, 100; B, 50.

**Fig. 7.**
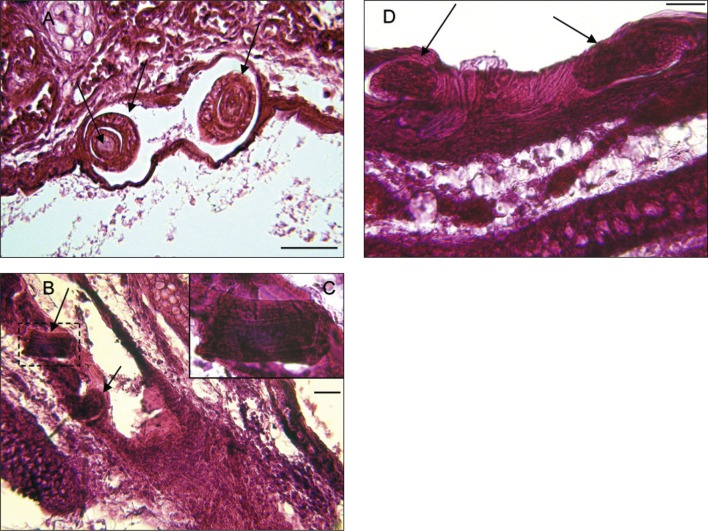
*Trichosomoides nasalis* (arrows) in the pseudostratified epithelium. A, two sections of a female (short arrows) with intrauterine male (long arrow). B, a female and inflammatory reaction. C, detail of an intrauterine male. D, female beneath the keratinized upper part of the pseudostratified epithelium which is stretched and distorted by worm movements. Scale bars in μm: A, D, 50; B, 40.

Thirty rodents were processed and examined. Sections of worms were found in nine of these. In one of six rodents fixed at 19 dpi, *T. nasalis* was found in the thorax. A single developing worm, 25 μm wide at the level of the stichocytes, was located in a striated muscle fibre of the intercostal muscles (Fig. 2). The remaining sections, in which a total of 33 *T. nasalis* were present, were restricted to region A of the maxilla (Fig. 1), including the muzzle, nasal vestibulum and the anterior part of the nasal cavities, where the anterior and median conchae are present (Fig. 3). The localization of these specimens in the tissue, their sex (based on body width, see below) and number, are presented in Table I and Figs 1-7.

**Table I.** Migration of *Trichosomoides nasalis* in intraperitoneally infected *Arvicanthis niloticus*: worm localization (n = 33) in the anterior part A of the maxilla (see Fig. 1).

Time post-infection^a^ (no. of infected hosts)	Worms recovered from infected hosts^b^	Worm diameter in μm (body level)	Sex	Infected host tissue		Figures	
19 dpi (n = 1)	1/1	29 (stichocytes)	f	blood vessel in the lamina propria		5B	
	2/1	33 (preoesophagus)	f	blood vessel in the lamina propria		5C	
	3/1	25 (preoesophagus)	f	lamina propria		-	
	4/1	34 (preoesophagus)	f	lamina propria		-	
	5/1	36 (preoesophagus)	f	lamina propria		-	
	6/1	37 (stichocytes)	f	pseudostratified epithelium		-	
	7/1	28 (stichocytes)	f	pseudostratified epithelium		-	

20 dpi (n = 5)	1/2	48 (uterus)/23^c^ (nd)	f/m	pseudostratified epithelium with inflammatoryreaction		7A	
	
	1/3	34 (nd)	f		dermal connective tissue of the nasal vestibulum	4D	
	2/3	30 (nd)	f		between muscle fibers of the nasal vestibulum	-	
	3/3	28 (nd)	f		between muscle fibers of the nasal vestibulum	4C	
	4/3	22 (nd)	m		lamina propria	5A	
	5/3	22 (nd)	m		lamina propria	-	
	6/3	40 (uterus)/20^c^ (nd)	f/m		pseudostratified epithelium with inflammatoryreaction	7B & 7C	
	7/3	40 (uterus)/20^c^ (nd)	f/m		pseudostratified epithelium	6C	
	
	1/4	42 (uterus)/20^c^ (nd)	f/m		pseudostratified epithelium	-	
	2/4	45 (uterus)/20^c^ (nd)	f/m		pseudostratified epithelium	7D	
	3/4	38 (stichocytes)	f		pseudostratified epithelium	-	
	4/4	35 (uterus)/18^c^ (nd)	f/m		pseudostratified epithelium	-	
	
	1/5	22 (nd)	m		between muscle fibers of the muzzle	4B	
	2/5	30 (stichocytes)	f		pseudostratified epithelium	-	
	
	1/6	28 (stichocytes)	f		muzzle connective tissue	3B	
	2/6	31 (gonad)	f		muzzle adipose tissue	3B & 4A	
	3/6	33(stichocytes)	f		pseudostratified epithelium	3B	
	4/6	33 (stichocytes)	f		pseudostratified epithelium	3A	
	5/6	35 (stichocytes)	f		pseudostratified epithelium	3A	
	6/6	34 (gonad)	f		pseudostratified epithelium	3A	

21 dpi (n = 2)	1/7	40 (uterus)/20^c^ (nd)	f/m		pseudostratified epithelium	-	
	2/7	35 (stichocytes)	f		pseudostratified epithelium	-	
	3/7	33 (stichocytes)	f		pseudostratified epithelium		-
	
	1/8	45 (uterus)/20^c^ (nd)	f/m		pseudostratified epithelium		-
	2/8	42 (uterus)/20^c^ (nd)	f/m		pseudostratified epithelium		-
	3/8	50 (uterus)/20^c^ (nd)	f/m		pseudostratified epithelium		-

a dpi: days post-infection;

b specimen reference number/host reference number;

c the first numeral refers to the diameter of the female, the second to the diameter of the male; f: female; f/m: female with intra-uterine male; m: male; nd: not determined.

In the tissues surrounding the nasal cavities, sections of *T. nasalis* were seen in the connective and adipose tissue of the muzzle, the dermal connective tissue of the nasal vestibulum, between muscle fibres of the nasal vestibulum and the external aspect of the maxilla (Fig. 4). In the nasal cavities (Figs 5-7), the worms were found in the connective tissue of the mucosa (lamina propria), in mucosal blood vessels, and in the pseudostratified epithelium. They did not occur in the mucous epithelium (Fig. 6).

Worms outside the epithelium of the nasal mucosa were found in rodents processed 19 and 20 dpi, whereas intra-epithelial worms were found from 19 dpi onwards. In the epithelium of the nasal mucosa, ten females contained intra-uterine males 20 and 21 dpi (Fig. 7). These males were 20-23 μm wide which is slightly smaller than observed during preceding morphological studies (Fall *et al.*, 2012), and is due to shrinkage during preparation for histological examination. Worms with a diameter more than 25 μm were considered as females. Three males were found free 20 dpi, one between muscles (Fig. 4B) and two in the lamina propria (Fig. 5A). None were identified free in the pseudostratified epithelium. Females were found outside and inside the nasal cavities in the lamina propria, its blood vessels, and pseudostratified epithelium. They stretched the comparatively thin epithelium in which some appeared coiled (Fig. 7D). Females containing males were only found in the epithelium. An acute inflammatory cell reaction was observed in the infected lamina propria (Fig. 7A & B).

## Discussion

Despite the fact that the prevalence and intensity of infection appeared to be lower in histological sections than the figures established during dissection, as one might expect, the total of 34 worms found, one from the thorax and 33 from the nose, were sufficient to establish the late migratory route of *T. nasalis* from the muscles to the nasal mucosa. Having completed their development, worms escape from the muscle fibres of the thoracic and abdominal walls (Fall *et al.*, 2012; Fig. 4). They subsequently move between muscles and through a variety of connective tissue towards the head, and enter the muzzle, nasal vestibulum and external aspect of the maxilla (Figs 3-4). They then reach the tissues of the nasal cavities where they are first found in the lamina propria, at 19 and 20 dpi (Table I). At 21 dpi, migration is completed and worms have reached their extremely specific definitive site, the region between the incisors, that is the epithelial lining of the anterior and median conchae, with an exclusive tropism for the pseudostratified epithelium. The migration of worms in the mucosa induces an inflammatory reaction, which develops into a rhinitis when the females mature (Diagne *et al.*, 2004). At this stage it is probable that the competitiveness of infected rodents with respect to search for food and possible mates would be reduced when compared to those not infected.

In the course of the present study it was also noted that three free males were not found in the epithelium, but rather in the lamina propria and between muscle fibres of the muzzle, whereas intra-uterine males were only seen in intra-epithelial females. Mating, or penetration of the male into the female, thus occurs when the female settles in the epithelium of the nasal mucosa. These observations support our previous hypothesis (Diagne *et al.*, 2004) that *T. nasalis* mates in the tissues and not in the lumen. Luminal mating was suggested for a parasite of the bladder, *Trichosomoides crassicauda* (Bellingham, 1840), by Thomas (1924) following dissection of experimentally infected rats, and for another trichosomoidin parasite of the paracloacal glands, *Anatrichosoma haycocki* Spratt, 1982, following dissections of naturally infected dasyurid marsupials (Spratt, 1982). Indeed, these authors found a few young adults in the ureters and small intestine, respectively. Interestingly, however, mature *A. haycocki* males were found in the epithelium of the glands and only gravid females were in the lumen of the paracloacal glands (Spratt, 1982). This suggests that *A. haycocki* also copulates in tissues.

The presence of migrating *T. nasalis* worms in blood vessels is worth comment. This localization is rare (two of 33 worms) and was observed only in the mucosa of the nasal cavities. The vascular system is well developed in the lamina propria (Figs 5B & C, 7A). The worms, highly motile during the final migration as seen at dissection, seem to force their way between the tissues, using their stylet which is still present, and very likely also secretions, as seen in *Trichinella spiralis* (Owen, 1835) (ManWarren *et al.*, 1997; Dzik, 2006). This indicates that they can, and do, enter blood vessels they encounter on their way to the epithelium.

Localization of adult worms in the vasculature is not a feature particular to *T. nasalis* and it has been observed in *T. crassicauda* by Thomas (1924) and other Trichosomoidinae, *Anatrichosoma cutaneum* (Swift, Boots & Miller, 1922) and *Anatrichosoma cynamolgi* Smith & Chitwood, 1954, both of which parasitize the nasal vestibulum of monkeys. In these two species male worms were found in the vessels of the lamina propria in histological sections (Allen, 1960; Long *et al.*, 1976). It is noteworthy that *Anatrichosoma* spp. males are as long as the females but much more slender, and do not live permanently in the uterus of the female. When copulating the male inserts half its body into the vulva of the female which is in the stratified epithelium, as observed with *Anatrichosoma buccalis* Pence & Little, 1972, a parasite of a didelphid marsupial (Little & Orihel, 1972). The vascular localization of males seen with parasites of monkeys might be a resting place between copulations assuming that males copulate more than once.

The biological data observed for *T. nasalis* has highlighted some important common features with *Trichinella* spp., like the muscle tropism of the hatching larvae. In *T. spiralis*, too, copulation occurs in the mucosal epithelium of the intestine (Gardiner, 1976). This might be a general primary feature.
